# Risk factors of recurrence in chronic subdural hematoma and a proposed extended classification of internal architecture as a predictor of recurrence

**DOI:** 10.1007/s10143-022-01790-8

**Published:** 2022-04-23

**Authors:** Hussam Hamou, Mohamed Alzaiyani, Rastislav Pjontek, Benedikt Kremer, Walid Albanna, Hani Ridwan, Hans Clusmann, Anke Hoellig, Michael Veldeman

**Affiliations:** 1 Department of Neurosurgery, RWTH Aachen University Hospital, Pauwelsstrasse 30, 52074 Aachen, Germany; 2 Department of Diagnostic and Interventional Neuroradiology, RWTH Aachen University, Aachen, Germany

**Keywords:** Chronic subdural hematoma, Recurrence, CT imaging, Classification, Internal architecture

## Abstract

Chronic subdural hematomas (cSDHs) constitute one of the most prevalent intracranial disease entities requiring surgical treatment. Although mostly taking a benign course, recurrence after treatment is common and associated with additional morbidity and costs. Aim of this study was to develop hematoma-specific characteristics associated with risk of recurrence. All consecutive patients treated for cSDH in a single university hospital between 2015 and 2019 were retrospectively considered for inclusion. Size, volume, and midline shift were noted alongside relevant patient-specific factors. We applied an extended morphological classification system based on internal architecture in CT imaging consisting of eight hematoma subtypes. A logistic regression model was used to assess the classification’s performance on predicting hematoma recurrence. Recurrence was observed in 122 (32.0%) of 381 included patients. Apart from postoperative depressed brain volume (OR 1.005; 95% CI 1.000 to 1.010; *p* = 0.048), neither demographic nor factors related to patient comorbidity affected recurrence. The extended hematoma classification was identified as a significant predictor of recurrence (OR 1.518; 95% CI 1.275 to 1.808; *p* < 0.001). The highest recurrence rates were observed in hematomas of the homogenous (isodense: 41.4%; hypodense: 45.0%) and sedimented (50.0%) types. Our results support that internal architecture subtypes might represent stages in the natural history of chronic subdural hematoma. Detection and treatment at a later stage of spontaneous repair can result in a reduced risk of recurrence. Based on their high risk of recurrence, we advocate follow-up after treatment of sedimented and homogenous hematomas.

## Introduction

Chronic subdural hematoma (cSDH) constitutes a distinct type of intracranial hemorrhage, prevalent among elderly patients. In the last decade, the incidence of cSDH has progressively increased reaching around 8 to 18 cases per 100,000 people per year. In patients 80 years or older, incidence increases up to 36.6/100,000 patients per year [1–3]. Population aging not only is a driving force behind incidence but also contributes to a growing number of patients under antithrombotic medication and other comorbidities upon presentation [4, 5].

The exact pathogenesis of cSDH remains debated although it has long been accepted that minor head injury causes a small venous subdural bleed originating from a bridging vein. Alternatively, traumatic separation of the dura-arachnoid junction and development of subdural hygroma has been postulated as the initial stage [6, 7]. In most cases, the acute subdural collection undergoes gradual resorption resulting in complete healing over time; however, in the presence of certain predisposing factors, liquefaction of the blood clot occurs with gradual enlargement of the subdural fluid collection. Causes of head trauma and predisposing pre-morbid factors overlap and include age, brain atrophy, epilepsy, antithrombotic treatment, and alcohol abuse [3]. The pathogenic cycle leading to cSDH formation can be regarded as a complex deranged repair mechanism which involves key processes such as fibrinolysis, neoangiogenesis, and inflammation [8]. In recent years, it has become clearer that damage to dural border cells plays a key role in the development of cSDH. During chronification, fibro-proliferation leads to build up of collagenous hematoma membranes consisting of an inner (visceral) layer overlapping the arachnoid surface of the brain, and outer (parietal) membrane covering the inner dura. The outer membrane receives its vascularization from meningeal arterial branches, and promising treatment results have been achieved by endovascular closure of the middle meningeal artery [9, 10].

Due to the direct relieve of mass effect, surgery remains the treatment of choice in symptomatic cases or patients showing increase in hematoma size. Trabecular hematoma membranes only rarely encapsulate and isolate hematoma compartments alleviating the need for larger craniotomies. Therefore, burr hole evacuation with irrigation and placement of a non-suction drainage has become the mainstay of treatment [11, 12]. Recurrence necessitating reoperation is a common complication associated with not only additional costs but also patient morbidity and decreased functional outcome [13]. Reoperation rates vary widely between 5 and 33% [3, 14] and identified predictors of recurrence are hematoma size, preoperative antithrombotic treatment, prior history of epileptic seizures, and persisting postoperative midline shift and brain re-expansion failure [15–19].

In recent year, it has become more accepted that changes within the hematoma’s architecture, and mainly the formation of membranes and septae, are the result of a spontaneous repair effort [3, 20]. During this process, volume expansion can decompensate resulting in clinical symptoms and diagnosis. The hypothesis of this study is that, based on the stage the hematoma is diagnosed in, the amount of natural healing already taken place can be estimated and might influence the recurrence rate. This concept was debuted by Nakaguchi et al. [21] who described four distinct hematoma types with an identified higher recurrence rate in less organized and presumably younger hematomas. This classification system has already been adopted in a scoring system for recurrence risk assessment [22]. Unfortunately, the existing classification into four hematoma types does not allow allocation of all cSDHs encountered in clinical practice. Moreover, the hypothesis that these subtypes represent stages in the natural disease history has not been previously tested. We postulate that an adjusted system might allow classification of all hematomas and provide a more refined recurrence risk estimation.

## Materials and methods

### Patient population and study design

All consecutive patients treated for cSDH in a single university hospital between 2015 and 2019 were retrospectively considered for inclusion. This study was approved by the local ethics committee (EK399/20) and was registered in the German Clinical Trials Register (DRKS00025280). The manuscript is written in accordance with the STROBE statement for reporting observational studies. Data were extracted from hospital records retrieving patients’ demographics, preoperative imaging hematoma characteristics, eventual recurrence, and postoperative imaging characteristics. Inclusion criteria were defined as chronic subdural hematomas as confirmed on CT imaging (uni- or bilateral) treated by surgical evacuation either via twist drill craniostomy, single or double burr hole craniotomy, or bone flap craniotomy. Exclusion criteria were (1) < 18 years of age, (2) prior causal neurosurgical or other potentially causal cranial procedures, (3) causal intracranial hypotension, i.e., shunt overdrainage and spinal dural leaks, and (4) non-iatrogenic coagulation disorders (e.g., liver failure and bleeding diathesis).

The following patient-specific factors were registered: age, gender, recall of trauma, documented trauma on CT imaging, presenting symptoms and/or neurological deficits, prior use of antithrombotic medication, preoperative comorbidities (e.g., hypertension, diabetes, and cancerous disease), and use of statins or ACE inhibitors.

### Standard treatment algorithm

After diagnosis, chronic subdural hematomas were treated when causing neurological deficit: paresis, gait disturbance, speech disorder, or seizures. Headache as an isolated symptom was not considered an indication for surgery. Asymptomatic hematomas were treated in case of clear mass effect reflected in imaging as either sulcal effacement, ventricle compression, and/or midline shift.

Treatment of choice was either twist drill craniostomy (TDC) without drainage or burr hole craniotomy (BHC) with placement of one or two non-suction subdural drains after irrigation of the hematoma cavity. Surgeon’s preference was the driving factor determining the type of initial surgery. A closed passive drainage system with silicon tubing was left behind in the subdural space and removed after 1 to 3 days as soon as they stopped draining. Bone flap craniotomy (BFC) was reserved for cases with an amount of acute blood (hyperintense on CT) not deemed removable via a BHC.

Routine postoperative imaging was performed for TDC cases but not after BHC. In the latter, imaging during the initial stay was only performed when new symptoms/deficits developed, or preoperative deficits persisted. If residual hematoma was identified, this would trigger revision surgery during the same hospital stay. Patients were discharged either with complete relieve of symptoms or with postoperative imaging revealing no space-occupying residual hematoma. After discharge, follow-up imaging was routinely performed 14 to 21 days post-surgery and in case of asymptomatic recurrence without mass effect, the hematoma was sequentially followed in 2-week intervals until healed or until progress caused symptoms or mass effect. Recurrence was defined as an increase in volume of residual or newly formed hematoma with mass effect due to midline shift, sulcal effacement, or new development or re-appearance of neurological symptoms (i.e., paresis, gait disturbance, and speech disorder), resulting in the need for reoperation. Follow-up with sequential CT imaging was continued until residual hematoma was resolved. No rigid cut-off value for volume defining recurrence was applied. Asymptomatic not space-occupying rest hematomas were followed postoperatively until full remission was documented on imaging. Small persisting asymptomatic no space-occupying hygromas in elderly patients with brain atrophy were also considered as full hematoma resolution, and were no longer followed. Recurrence after 6 months, with documented remission in between, was considered as new disease.

### Clinical and radiological assessment

For preoperative radiological assessment, hematoma dimensions were measured in length along the longest axis (mm), width at its widest (mm), and volume (ml) was software assisted reconstructed (Brainlab, Munich, Germany). For bilateral hematomas, the largest dimension was used. The presence of midline shift and its extension (mm) was noted. Each hematoma was classified according to one of four Nakaguchi subtypes (homogenous, laminar, separated, trabecular type) as described in the original publication [21] by two independent assessors (HH and MV) blinded to additional information. Based on the initial categorization by both assessors, Cohen’s kappa (*κ*) was calculated to quantify inter-rater reliability. Incongruent results were discussed upon reaching unequivocal consensus categorization for further analysis. Postoperative depressed brain volume (ml) was also software assisted measured out (Brainlab, Munich, Germany) in patients with early postoperative imaging available. Depressed brain volume includes all supratentorial intracranial volume not filled with re-expanded brain, e.g., residual hematoma, rinsing fluid, and air. The delay in days between surgery and imaging, on which depressed brain volume was measured, was noted.

**Fig. 1:**
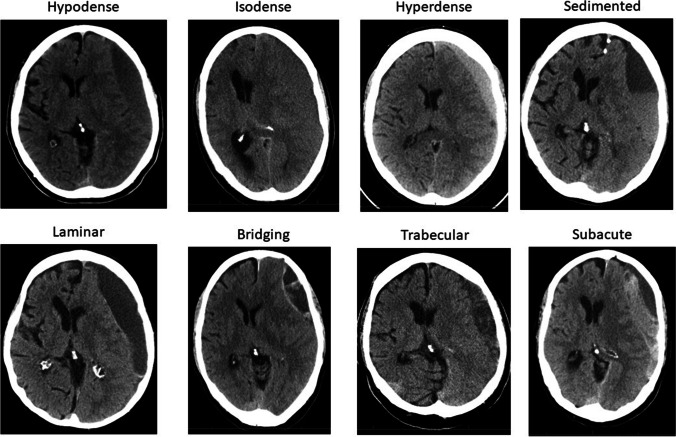
Overview of all eight hematoma subtypes within the extended classification, based on internal architecture as seen in CT imaging

**Fig. 2:**
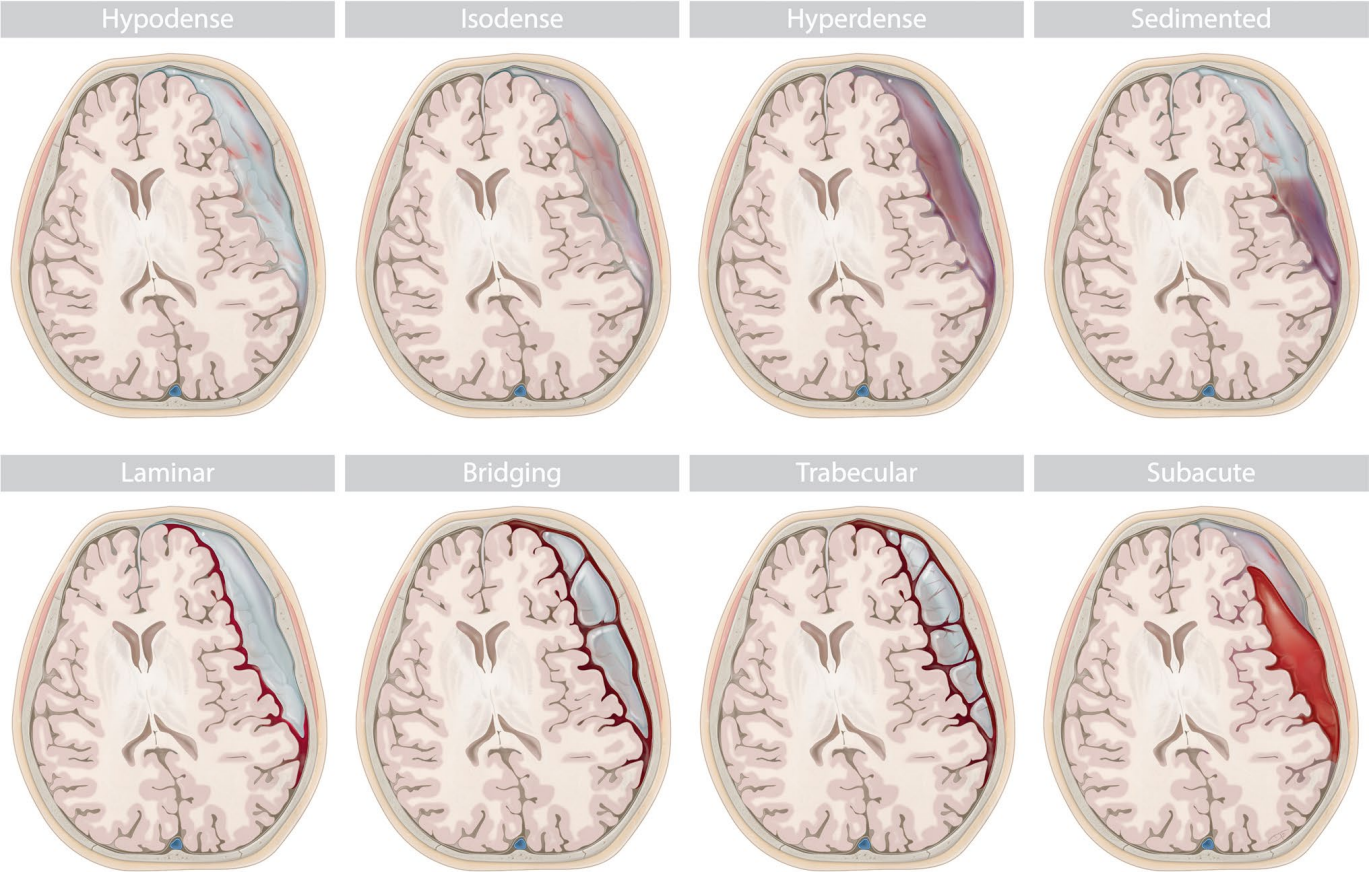
Stereotypic depiction of eight hematoma subtypes

Additionally, the Nakaguchi classification was extended to accommodate all encountered hematomas. We defined eight distinct subtypes based on hematoma density and internal architecture as seen in CT imaging (Fig. 1). The homogenous and trabecular types were subdivided into each three distinct entities and a subacute type was added: (1) homogenous hypodense: consisting of homogenous hematoma appearing hypodense compared to brain tissue; (2) homogenous isodense: consisting of homogenous hematoma isodense compared to brain tissue; (3) homogenous hyperdense: consisting of homogenous hematoma hyperdense in relation to brain tissue with lack of immediate history of head trauma; (4) sedimented: where hemoglobin sediments are separated from less dense content by gravity during imaging; (5) laminar: where a hyperdense visceral or parietal membrane is visible; (6) bridging: where a countable number of internal membranes connect the visceral and parietal membrane; (7) trabecular type: with a more complex and diffuse membrane structure precluding the counting of membranes; (8) subacute: chronic subdural hematoma with small acute blood components with or without visceral/parietal membranes and lack of immediate history of head trauma. A stereotypical representation of hematoma types is depicted in Fig. 2. All hematomas were classified independently by two assessors (HH and MV), as described above. For all classifications, if morphological features of multiple subtypes were present in a single hematoma, the more organized subtype was allocated.

The primary outcome was defined as the effect of the extended hematoma classification on recurrence as analyzed in logistic regression. Secondary outcome parameters were the effects on recurrence of initial clinical presentation, comorbidity (hypertension, diabetes, cancerous disease, alcohol abuse), prior use of ACE inhibitor or statins, the prior use of antithrombotics hematoma size (length, width, and volume), and postoperative depressed brain volume. Additionally, a subgroup of cases with documented cranial trauma in CT imaging, preceding the development of symptomatic cSDH, was identified.

### Statistical analysis

All data are presented as mean and standard deviation for normally, and as median and interquartile rage (Q_1_–Q_3_) for non-normally distributed continuous variables. Nominal and ordinal categorical variables are provided as frequencies or proportions. Data were tested for normality via the Shapiro–Wilk test, and based on the results, the appropriate univariate statistical test was selected. Nominal data were tested with the chi-square test and continuous data via the unpaired *t*-test if normally, and the Mann–Whitney *U*-test, if not normally distributed. A logistic regression model was build introducing all univariate factors with a *p*-value < 0.15 [23]. Prior to factor inclusion, variables were tested for outliers via plotting, and multicollinearity was evaluated via the assessment of the variance inflation factor with a cut-off at 2.5. Multiple groups of non-parametric continuous data were compared in a Kruskal–Wallis test. Inter-rater reliability in appointing hematoma subtypes was measured by calculation of Cohen’s kappa (*κ*). Missing data was not imputed. All statistical analyses were performed using IBM SPSS Statistics 25 (SPSS Inc., Chicago, IL, USA). Statistical significance was defined as a two-sided *p* < 0.05.

## Results

### Patients

Based on hospital records, 408 patients were surgically treated for chronic subdural hematoma during the inclusion time frame. A total of 27 cases were excluded based on the following reasons: 12 cases with predominantly acute hematomas, 10 cases because preoperative imaging was no longer available, and 5 cases with related prior cranial surgery. Of the resulting 381 patients (137 women and 244 men) with a mean age of 75.2 ± 12.0, a total of 92 (24.1%) had bilateral cSDH. Burr hole craniotomy with subdural drainage was the most common performed technique (250 case; 65.6%) before TDC (121 cases; 31.8%). Primary BFC was performed in 10 cases (2.6%) of which two were of the homogenous hypodense, one was of the laminar, one was of the trabecular, and six were of the subacute type. Redo surgery during the initial hospital stay because of incomplete hematoma evacuation after TDC was necessary in 52 (43.0%) patients.

### Recurrence

Recurrence as defined above was observed in 122 (32.0%) patients. The highest recurrence rates were noted in the homogenous (hypodense: 45.0%; isodense: 41.4%) and sedimented (50.0%) hematoma types (Table 1). Based on univariate analysis, 9 variables were eligible to be introduced as predictors in the regression model: gait disturbance as the presenting symptom (*p* = 0.093); preoperative epilepsy (*p* = 0.112); hematoma width (*p* = 0.117); hematoma length (*p* = 0.083); hematoma volume (*p* = 0.002); midline shift (*p* = 0.090); postoperative depressed brain volume (*p* = 0.002); the Nakaguchi classification (*p* < 0.001); and the extended hematoma classification (*p* < 0.001) (Table 2). Due to collinearity between hematoma width, length, volume, and midline shift, only hematoma width was introduced into the model. Width was chosen, as it is rapidly reproducible and does not require additional software. Additionally, a significant positive correlation between hematoma width and midline shift was confirmed in this cohort (Spearman’s *ρ* = 0.192; *p* = 0.001). The binomial logistic regression model was run with each of both hematoma classifications separately.

**Table 1:** Recurrence rates and time to develop for both internal architectural classification systems for chronic subdural hematoma

Nakaguchi classification — no. (%)	All (*n* = 381)	No recurrence (*n* = 259)	Recurrence (*n* = 122)	Recurrence rate	Average time to develop in days (median (Q_1_–Q_3_)) (*n* = 51)	*p*-value*
Trabecular	107 (28.1)	89 (34.4)	18 (14.8)	16.8%	56 (42–90)	0.012
Laminar	58 (15.2)	45 (17.4)	13 (10.7)	22.4%	16 (12–19)	
Homogenous	189 (49.6)	111 (42.9)	78 (63.9)	41.3%	31 (20–47)	
Separated	27 (7.1)	14 (5.4)	13 (10.7)	48.1%	n/a	
Extended classification — no. (%)						
Bridging	35 (9.2)	32 (12.4)	3 (2.5)	8.6%	62 (58–77)	0.551
Subacute	27 (7.1)	23 (8.9)	4 (3.3)	14.8%	48 (23–76)	
Laminar	45 (11.8)	38 (14.7)	7 (5.7)	15.6%	19 (12–46)	
Trabecular	61 (16.0)	45 (17.4)	16 (13.1)	26.2%	32 (20–46)	
Hyperdense	20 (5.2)	13 (5.0)	7 (5.7)	35.0%	32 (30–41)	
Isodense	87 (22.8)	51 (19.7)	36 (29.5)	41.4%	16 (13–38)	
Hypodense	80 (21.0)	44 (17.0)	36 (29.5)	45.0%	33 (23–45)	
Sedimented	26 (6.8)	13 (5.0)	13 (10.7)	50.0%	n/a	

*n/a*, not available; *Q*_*1*_*–Q*_*3*_, first quartile–third quartile

^*^Results of Kruskal–Wallis test for development times for each subtype of the 51 patients with trauma documented by CT imaging

**Table 2:** Comparison of patient-, hematoma-, and surgery-specific characteristics in patient with or without hematoma recurrence. In univariate analysis, nine initial variables (*) were identified as potential predictors to be introduced into the logistic regression model

	All (*n* = 381)	No recurrence (*n* = 259)	Recurrence (*n* = 122)	*p*-value
Demographics				
Age — mean ± SD	75.2 ± 12.0	75.5 ± 12.4	74.7 ± 11.3	0.312
Gender — F (%)/M (%)	137 (36.0)/244 (64.0)	95(36.7)/164 (63.3)	42 (34.4)/80 (65.6)	0.669
Initial presentation				
Initial GCS–median [Q_1_–Q_3_]	15 [14–15]	15 [14–15]	15 [14–15]	0.719
Preoperative deficit — no. (%)				
Neurological deficit	354 (92.9)	243 (93.8)	111 (91.0)	0.351
Aphasia	81 (21.3)	57 (22.0)	24 (19.7)	0.618
Paresis	199 (52.2)	139 (53.7)	60 (49.2)	0.437
Gait disturbance	145 (38.1)	106 (40.9)	39 (32.0)	0.093*
Preoperative epilepsy	21 (5.5)	11 (4.2)	10 (8.2)	0.112*
Comorbidity				
Arterial hypertension—no. (%)	226 (59.3)	159 (61.4)	67 (54.9)	0.230
Arrythmias—no. (%)	91 (23.9)	64 (24.7)	27 (22.1)	0.610
CAD—no. (%)	132 (34.6)	94 (36.3)	38 (31.1)	0.325
Diabetes—no. (%)	71 (18.9)	50 (19.3)	21 (17.2)	0.625
Cancerous disease—no. (%)	59 (15.5)	40 (15.4)	19 (15.6)	0.948
Alcohol abuse—no. (%)	18 (4.7)	13 (5.0)	5 (4.1)	0.705
Illicit drug use—no. (%)	7 (1.8)	5 (1.9)	2 (1.6)	0.851
Prior MI or stroke—no. (%)	61 (16.0)	43 (16.6)	19 (15.6)	0.899
Prior medication—no. (%)				
ACE inhibitors	131 (34.4)	87 (33.6)	44 (36.1)	0.635
Statins	83 (21.8)	58 (22.4)	25 (20.5)	0.675
Antiplatelet	107 (28.1)	29 (23.8)	78 (30.1)	0.191
Anticoagulant	43 (11.3)	11 (9.0)	32 (12.4)	0.325
Hematoma characteristics				
Bilateral—no. (%)	92 (24.1)	60 (23.2)	32 (26.2)	0.514
Width (mm)—mean ± SD	22.1 ± 5.8	21.8 ± 5.7	22.8 ± 5.9	0.117*
Length (mm)—mean ± SD	123.8 ± 59.5	122.9 ± 71.2	125.7 ± 21.5	0.083*
Volume (ml)—mean ± SD	147.9 ± 134.0	145.25 ± 160.3	153.1 ± 48.0	0.002*
Midline shift—no. (%)	252 (66.1)	164 (63.3)	88 (72.1)	0.090*
MLS (mm)—mean ± SD	9.2 ± 4.0	9.2 (3.8)	9.3 (4.3)	
Hematoma evacuation				0.651
Twist drill craniostomy	121 (31.8%)	79 (65.3)	42 (34.7)	
Burr hole craniotomy	250 (65.6)	173 (69.2)	77 (30.8)	
Bone flap craniotomy	10 (2.6)	7 (70.0)	3 (30.0)	
Internal architecture				
Nakaguchi type—no. (%)				< 0.001*
Homogenous	189 (49.6)	111 (42.9)	78 (63.9)	
Laminar	58 (15.2	45 (17.4)	13 (10.7)	
Separated	27 (7.1)	14 (5.4)	13 (10.7)	
Trabecular	107 (28.1)	89 (34.4)	18 (14.8)	
Extended type—no. (%)				< 0.001*
Homogenous hypodense	80 (21.0)	44 (17.0)	36 (29.5)	
Homogenous isodense	87 (22.8)	51 (19.7)	36 (29.5)	
Homogenous hyperdense	20 (5.2)	13 (5.0)	7 (5.7)	
Sedimented	26 (6.8)	13 (5.0)	13 (10.7)	
Laminar	45 (11.8)	38 (14.7)	7 (5.7)	
Bridging	35 (9.2)	32 (12.4)	3 (2.5)	
Trabecular	61 (16.0)	45 (17.4)	16 (13.1)	
Subacute	27 (7.1)	23 (8.9)	4 (3.3)	
Early postoperative imaging (*n* = 216)				
Days after surgery–median [IQR]	1 [1–3]	2 [1–3]	1 [1–3]	0.089
Depressed brain volume (ml)–median [Q_1_–Q_3_]	77.0 [45.2–116.3]	70.7 [42.3–100.0]	95.5 [64.3–150.2]	0.002*

*ACE*, angiotensin-converting enzyme; *CAD*, coronary arterial disease; *F*, female; *GCS*, Glasgow coma scale; *M*, male; *MI*, myocardial infarction; *mm*, millimeter; *Q*_*1*_*–Q*_*3*_, first quartile–third quartile; *SD*, standard deviation

In the extended classification, the prevalence of recurrence was highest for the sedimented type (50.0%) and lowest for hematomas with a bridging architecture (8.6%) (Table 1). Hematoma types were introduced in the model categorized according to increasing prevalence of recurrence. The logistic regression model was statistically significant (*χ*^2^(5) = 30.546, *p* < 0.001). The model explained 18.6% (Nagelkerke *R*^2^) of the variance in recurrence and was able to classify 71.7% of cases. Of the predictor variables, only three were statistically significant: postoperative depressed brain volume (OR 1.005; 95% CI 1.000 to 1.010; *p* = 0.048) and both the Nakaguchi (OR 2.397; 95% CI 1.646 to 4.491; *p* < 0.001) and extended hematoma classification (OR 1.518; 95% CI 1.275 to 1.808; *p* < 0.001) (Table 3).

**Table 3:** Analysis in a logistic regression model of three selected predictor variables in conjunction with both hematoma classification systems

	No recurrence (*n* = 259)	Recurrence (*n* = 122)	OR	95% CI	*p*-value
Gait disturbance—no. (%)	106 (40.9)	39 (32.0)	1.116	0.577–2.158	0.744*
Preoperative epilepsy—no. (%)	11 (4.2)	10 (8.2)	0.977	0.291–3.278	0.969*
Width (mm)—mean ± SD	21.8 ± 5.7	22.8 ± 5.9	1.042	0.987–1.101	0.136*
Postoperative depressed brain volume (ml)—median [Q_1_–Q_3_]	70.7 [42.3–100.0]	95.5 [64.3–150.2]	1.005	1.000–1.010	0.048*
Nakaguchi classification—no. (%)			2.397	1.646–4.491	** < **0.001^#^
Extended classification—no. (%)			1.518	1.275–1.808	** < **0.001*

^*^Results of logistic regression with the extended classification as the fifth predictor variable in the model

^#^Results of the logistic regression with the Nakaguchi classification as the fifth predictor variable in the model

*CI*, confidence interval; *mm*, millimeter; *OR*, odds ratio; *SD*, standard deviation

Noteworthy is that patients undergoing redo surgery after TDC during the initial hospital stay presented with a higher risk of recurrence after discharge (29 (11.2%) *versus* 23 (18.9%); *p* = 0.042). This effect could be explained by the fact that most patients initially treated with TDC had hematomas of the homogenous 26 (50.0%) or sedimented 6 (11.5%) type.

### Inter-rater reliability

A Cohen’s *κ* of 0.763 (*p* < 0.001) was calculated for the Nakaguchi classification. The highest observer agreement was identified for the homogenous type with 88.9% of hematomas classified as homogenous by both assessors. Congruence was the lowest for the laminar subtype with only 70.7% of classifications matching between assessors. The highest rate of mismatching was identified between the homogenous and laminar type with 19.0% of cases being mismatched.

A Cohen’s *κ* of 0.835 (*p* < 0.001) was calculated for the extended classification. The highest rate of inter-rater matched results was found for the sedimented type with 96.2% of hematomas classified as sedimented by both assessors and was the lowest for the homogenous hyperdense type, of which only 70.0% of the assigned subtype matched between raters. The highest rate of mismatching was observed between the bridging and trabecular subtypes (14.8%).

### Documented trauma

In 51 patients, CT imaging of a documented trauma preceding the development of cSDH was available. Only in 10 cases (19.6%), a small acute subdural hematoma was visible on initial CT scanning. There were no documented trauma cases who presented with a sedimented type upon diagnosis. It took a median of 31 (17–52) days after the initial trauma to develop symptomatic hematomas. Hematomas of the bridging type took the longest to develop (median of 62 days (58–77)) compared to homogenous isodense hematomas (median of 16 days (13–38)) who took the least amount of time to become symptomatic. However, potentially due to small group sizes for each individual hematoma type, development times did not differ significantly between hematoma types (*p* = 0.551) (Table 1).

## Discussion

This study constitutes an effort to develop a more detailed classification of chronic subdural hematoma, allowing more precise recurrence risk stratification. The underlying hypothesis is that progressive fibro-proliferation within the hematoma constitutes a repair mechanism to close the artificially formed cavity.

Congruous with this hypothesis, homogenous hematomas presented with higher recurrence rates compared to more organized subtypes. In the latter, the more developed stage of natural healing reached until diagnosis might be protective against recurrence. Meaning that once the problem of mass effect is resolved by surgical evacuation, the more advanced natural repair aids in definitive hematoma resolution. It remains unclear where the sedimented/separated subtype — of which 50% reoccur — fits into this model of natural history. Nakaguchi et al. postulated that this subtype constitutes a stage after the laminar type and is as an outing of internal fibrinolysis. We prefer to consider the separated type as a subtype within the homogenous stages in which systemic or local dysfunctional coagulation fails to form a structurally supporting fibrin network causing a sediment when lying down during CT imaging. The high recurrence rates observed for this type can be explained by dysfunctional local coagulation [24].

Of all patient-specific factors, only internal architecture postoperative depressed brain volume was significantly associated with recurrence. Here, a decrease in one of eight levels of hematoma organization in the extended classification was associated with a 1.518-times increase in recurrence risk. Although we calculated a 2.397-fold risk increase for the Nakaguchi classification, this was spread out over four levels of hematoma organization. The eight subtypes within the extended classification allow for a more precise and adequate recurrence risk estimation compared to the Nakaguchi and more recently published classification systems [25]. Inter-rater reliability in imaging classification is often high but proved lower in the eight-subtype extended classification compared to the Nakaguchi classification. The homogenous type in the Nakaguchi system and the sedimented type in the extended classification proved to be the easiest to denote and presented with the least amount of mismatch between assessors. The laminar subtype of the Nakaguchi classification and the homogenous hyperdense subtype in the extended classification, on the other hand, presented with the highest inter-rater mismatch. Especially, differentiating between the bridging and trabecular subtypes presented with inter-rater variability due to subjective interpretation of the definition of these subtypes.

Our extended classification system requires additional external validation and reassessment of inter-rater reliability, to be able to extrapolate our results. Hematoma size, reflected by width, can be seen as an indirect measurement of brain compliance. Congruous to our results, the Nakaguchi classification and postoperative volume were significantly associated with recurrence, in a similar series of 208 cases [15]. In a series of 105 patients, preoperative hematoma width and more homogenous hematomas without inner membranes were also associated with higher recurrence rates [16].

When looking at patients with documented trauma, it took somewhat more than 4 weeks to develop a symptomatic hematoma. Based on our hypothesis of gradual hematoma transformation, we would expect longer development times for more organized types. This was not confirmed by our data, probably because hematoma subgroups of documented trauma cases were too small to draw any meaningful conclusion concerning time to develop for each subtype. As only a minority of cases had visible acute hemorrhage in the initial CT imaging, this underlines the importance of damage to the dural border cells and not the acute blood collection in initiating hematoma formation.

Schucht et al. advocate refraining from routine postoperative CT imaging after treatment of chronic subdural hematoma [26]. They base their argument on the fact that routine follow-up did not result in improvement of clinical outcome as measured by the modified Rankin scale after 6 months. It seems that waiting until new symptoms develop is sufficient to identify those patients requiring imaging and revision surgery. However, patients suffering from chronic subdural hematoma are not always in a state well enough to indicate their symptoms early. The risk of permanent neurological deficit due to late symptom recognition did not result in worse clinical outcome in their trial. Still, with a recurrence rate of 50%, we would recommend routine follow-up of patients treated for separated/sedimented hematoma types and even homogenous types. The approach is feasible to identify hematoma recurrence and growth early before onset of symptoms reducing the burden of this disease for the individual patient.

### Limitation

Being a retrospective designed monocentric study, our data is subjected to selection and performance bias. Selection bias was controlled by rigid in- and exclusion criteria of consecutive patients. Most patients were treated with burr hole surgery and closed-system drainage. However, many homogenous hematomas were initially evacuated via twist drill craniostomy. The choice of type of primary surgery was up to the surgeon’s preference introducing performance bias. As the success rate of the twist drill procedure is limited [27], it is a possible contributor to the higher recurrence rate of homogenous hematomas. However, this bias is partly corrected as patients treated via twist drill surgery routinely received postoperative imaging and were, in case of relevant residual hematoma, re-operated via burr hole craniotomy during the initial hospital stay. Incomplete removal of hematoma via the twist drill procedure requiring additional burr hole surgery was not classified as recurrence. On the other hand, routine post op imaging after burr hole craniotomy was not performed, and there is a remaining chance that recurrence on imaging later on is the result of failure of initial surgery to relieve mass effect. Additionally, bone flap craniotomy was more commonly performed in the subacute type of the extended classification and this treatment might have contributed to the low observed recurrence rate observed. Finally, other treatment algorithms, such as burr hole drainage without irrigation [28] or placement of subperiostal drains [29], were not assessed in this study.

## Conclusion

Internal architecture represents possible stages in the natural history and endogenous repair and natural history of chronic subdural hematomas. The proposed classification system contributes to a more precise recurrence risk stratification. Larger hematoma size, reflected by increasing width as well as decreasing internal organization, is associated with higher risk of recurrence. Homogenous and especially sedimented hematoma types have high recurrence rates and constitute the most useful subgroup for closer postoperative follow-up.

## Data Availability

The raw data on which this study and the analyses are based upon can be made available to qualified researchers on reasonable request.

## References

[1] Rauhala M, Luoto TM, Huhtala H, Iverson GL, Niskakangas T, Öhman J, Helén P (2019) The incidence of chronic subdural hematomas from 1990 to 2015 in a defined Finnish population. J Neurosurg 1–11 10.3171/2018.12.JNS183035 30901751

[2] Rauhala M, Helén P, Huhtala H, Heikkilä P, Iverson GL, Niskakangas T, Öhman J, Luoto TM (2020). Chronic subdural hematoma-incidence, complications, and financial impact. Acta Neurochir.

[3] Kolias AG, Chari A, Santarius T, Hutchinson PJ (2014). Chronic subdural haematoma: modern management and emerging therapies. Nat Rev Neurol.

[4] Toi H, Kinoshita K, Hirai S, Takai H, Hara K, Matsushita N, Matsubara S, Otani M, Muramatsu K, Matsuda S, Fushimi K, Uno M (2018). Present epidemiology of chronic subdural hematoma in Japan: analysis of 63,358 cases recorded in a national administrative database. J Neurosurg.

[5] Abe Y, Maruyama K, Yokoya S, Noguchi A, Sato E, Nagane M, Shiokawa Y (2017). Outcomes of chronic subdural hematoma with preexisting comorbidities causing disturbed consciousness. J Neurosurg.

[6] Lee KS (2004). Natural history of chronic subdural haematoma. Brain Inj.

[7] Kristof RA, Grimm JM, Stoffel-Wagner B (2008). Cerebrospinal fluid leakage into the subdural space: possible influence on the pathogenesis and recurrence frequency of chronic subdural hematoma and subdural hygroma. J Neurosurg.

[8] Edlmann E, Giorgi-Coll S, Whitfield PC, Carpenter KLH, Hutchinson PJ (2017). Pathophysiology of chronic subdural haematoma: inflammation, angiogenesis and implications for pharmacotherapy. J Neuroinflammation.

[9] Fiorella D, Arthur AS (2019). Middle meningeal artery embolization for the management of chronic subdural hematoma. J Neurointerv Surg.

[10] Moshayedi P, Liebeskind DS (2020). Middle meningeal artery embolization in chronic subdural hematoma: implications of pathophysiology in trial design. Front Neurol.

[11] Cenic A, Bhandari M, Reddy K (2005). Management of chronic subdural hematoma: a national survey and literature review. Can J Neurol Sci.

[12] Berghauser Pont LM, Dippel DW, Verweij BH, Dirven CM, Dammers R (2013). Ambivalence among neurologists and neurosurgeons on the treatment of chronic subdural hematoma: a national survey. Acta Neurol Belg.

[13] Santarius T, Kirkpatrick PJ, Ganesan D, Chia HL, Jalloh I, Smielewski P, Richards HK, Marcus H, Parker RA, Price SJ, Kirollos RW, Pickard JD, Hutchinson PJ (2009). Use of drains versus no drains after burr-hole evacuation of chronic subdural haematoma: a randomised controlled trial. Lancet (London, England).

[14] Weigel R, Schmiedek P, Krauss JK (2003). Outcome of contemporary surgery for chronic subdural haematoma: evidence based review. J Neurol Neurosurg Psychiatry.

[15] Ridwan S, Bohrer AM, Grote A, Simon M (2019). Surgical treatment of chronic subdural hematoma: predicting recurrence and cure. World Neurosurg.

[16] Yamamoto H, Hirashima Y, Hamada H, Hayashi N, Origasa H, Endo S (2003). Independent predictors of recurrence of chronic subdural hematoma: results of multivariate analysis performed using a logistic regression model. J Neurosurg.

[17] Chon KH, Lee JM, Koh EJ, Choi HY (2012). Independent predictors for recurrence of chronic subdural hematoma. Acta Neurochir.

[18] Miah IP, Tank Y, Rosendaal FR, Peul WC, Dammers R, Lingsma HF, den Hertog HM, Jellema K, van der Gaag NA (2020) Radiological prognostic factors of chronic subdural hematoma recurrence: a systematic review and meta-analysis. Neuroradiology 10.1007/s00234-020-02558-x PMC7803717 33094383

[19] Jang KM, Choi HH, Mun HY, Nam TK, Park YS, Kwon JT (2020). Critical depressed brain volume influences the recurrence of chronic subdural hematoma after surgical evacuation. Sci Rep.

[20] Edlmann E, Giorgi-Coll S, Whitfield PC, Carpenter KLH, Hutchinson PJ (2017). Pathophysiology of chronic subdural haematoma: inflammation, angiogenesis and implications for pharmacotherapy. J Neuroinflamm.

[21] Nakaguchi H, Tanishima T, Yoshimasu N (2001). Factors in the natural history of chronic subdural hematomas that influence their postoperative recurrence. J Neurosurg.

[22] Stanišic M, Pripp AH (2017). A reliable grading system for prediction of chronic subdural hematoma recurrence requiring reoperation after initial burr-hole surgery. Neurosurgery.

[23] Bursac Z, Gauss CH, Williams DK, Hosmer DW (2008). Purposeful selection of variables in logistic regression. Source Code Biol Med.

[24] Nomura S, Kashiwagi S, Fujisawa H, Ito H, Nakamura K (1994). Characterization of local hyperfibrinolysis in chronic subdural hematomas by SDS-PAGE and immunoblot. J Neurosurg.

[25] Takei J, Hirotsu T, Hatano K, Ishibashi T, Inomata T, Noda Y, Morooka S, Murayama Y (2021). Modified computed tomography classification for chronic subdural hematoma features good interrater agreement: a single-center retrospective cohort study. World Neurosurg.

[26] Schucht P, Fischer U, Fung C, Bernasconi C, Fichtner J, Vulcu S, Schöni D, Nowacki A, Wanderer S, Eisenring C, Krähenbühl AK, Mattle HP, Arnold M, Söll N, Tochtermann L, Z’Graggen W, Jünger ST, Gralla J, Mordasini P, Dahlweid FM, Raabe A, Beck J (2019). Follow-up computed tomography after evacuation of chronic subdural hematoma. N Engl J Med.

[27] Jablawi F, Kweider H, Nikoubashman O, Clusmann H, Schubert GA (2017). Twist drill procedure for chronic subdural hematoma evacuation: an analysis of predictors for treatment success. World Neurosurg.

[28] Uda H, Nagm A, Ichinose T, Onishi Y, Yoshimura M, Tsuruno T, Ohata K (2020). Burr hole drainage without irrigation for chronic subdural hematoma. Surg Neurol Int.

[29] Zhang JJY, Wang S, Foo ASC, Yang M, Quah BL, Sun IS, Ng ZX, Teo K, Pang BC, Yang EW, Lwin S, Chou N, Low SW, Yeo TT, Santarius T, Nga VDW (2019). Outcomes of subdural versus subperiosteal drain after burr-hole evacuation of chronic subdural hematoma: a multicenter cohort study. World Neurosurg.

